# Construction and characterization of an infectious clone of coxsackievirus A16

**DOI:** 10.1186/1743-422X-8-534

**Published:** 2011-12-13

**Authors:** Fei Liu, Qingwei Liu, Yicun Cai, Qibin Leng, Zhong Huang

**Affiliations:** 1 Key Laboratory of Molecular Virology & Immunology, Institute Pasteur of Shanghai, Shanghai Institutes for Biological Sciences, Chinese Academy of Sciences, 411 Hefei Road, Shanghai 200025, China

**Keywords:** Coxsackievirus A16, Infectious cDNA clone, *In vitro *transcription, Recovered virus

## Abstract

**Background:**

Coxsackievirus A16 (CVA16) is a member of the *Enterovirus *genus of the *Picornaviridae *family and it is a major etiological agent of hand, foot, and mouth disease (HFMD), which is a common illness affecting children. CVA16 possesses a single-stranded positive-sense RNA genome containing approximately 7410 bases. Current understanding of the replication, structure and virulence determinants of CVA16 is very limited, partly due to difficulties in directly manipulating its RNA genome.

**Results:**

Two overlapping cDNA fragments were amplified by RT-PCR from the genome of the shzh05-1 strain of CVA16, encompassing the nucleotide regions 1-4392 and 4381-7410, respectively. These two fragments were then joined *via *a native *Xba*I site to yield a full-length cDNA. A T7 promoter and poly(A) tail were added to the 5' and 3' ends, respectively, forming a full CVA16 cDNA clone. Transfection of RD cells *in vitro *with RNA transcribed directly from the cDNA clone allowed the recovery of infectious virus in culture. The CVA16 virus recovered from these cultures was functionally and genetically identical to its parent strain.

**Conclusions:**

We report the first construction and characterization of an infectious cDNA clone of CVA16. The availability of this infectious clone will greatly enhance future virological investigations and vaccine development for CVA16.

## Background

Coxsackievirus A16 (CVA16) and enterovirus 71 (EV71) are major etiological agents of hand, foot, and mouth disease (HFMD), which is a common illness in children [1-6]. Surveillance data indicate that CVA16 and EV71 often co-circulate during HFMD outbreaks [1-3,5-8]. The illness caused by CVA16 infection is usually mild [9], whereas EV71 infection is often associated with severe complications such as brainstem encephalitis, severe pulmonary edema and shock, and significant mortality [6,10,11]. Therefore, EV71 has been the main focus of virological investigations and vaccine development for HFMD. However, recent reports suggest that humans can be co-infected by CVA16 and EV71, and carry these two viruses simultaneously [12,13]. This co-infection may have contributed to the recently observed recombination between CVA16 and EV71 [14,15], which is believed to have led to the emergence of a recombinant EV71 responsible for the large HFMD outbreak in Fuyang City, China, during 2008 [15]. Furthermore, CVA16 infection is not always benign because fatal cases associated with CVA16 infection have been reported [16-18]. These findings indicate the significant importance of further investigation of CVA16 in order to understand better and ultimately control infections with this virus.

Both CVA16 and EV71 are members of the *Enterovirus *genus of the *Picornaviridae *family and they possess a single-stranded positive-sense RNA genome containing approximately 7400 bases. The CVA16 genome can be divided into 5'-non-coding, protein coding, and 3'-non-coding regions [19]. The 5'-non-coding region is ~740 nucleotides in length and it contains genetic elements required for genome replication and translation, for example, an internal ribosome entry site (IRES). The 3'-non-coding region is ~100 nucleotides in length and it is followed by a 3' poly(A) tail. The protein coding region consists solely of a single open reading frame that encodes a large polyprotein containing structural (P1) and non-structural (P2 and P3) regions [19]. Recent efforts have been directed toward the understanding of the expression, processing, and function of CVA16-encoded proteins. For example, the use of a panel of polyclonal antibodies against the recombinant capsid subunit proteins of CVA16 demonstrated that P1 can be processed by CVA16-encoded proteases to yield the subunit proteins VP0, VP1 and VP3, all of which subsequently co-assemble to form viral capsids [20]. However, further dissection and characterization of the role of individual viral proteins and genetic elements has been hindered by the difficulty of directly manipulating the RNA genome of CVA16.

For many RNA viruses, cDNA clones of the entire viral genome can serve as a template for the generation of infectious RNA. These infectious cDNA clones provide a platform for the manipulation of viral genomes and they provide a valuable tool for studying the molecular biology of virus replication, virus structure, virulence determinants, and vaccine development. Infectious cDNA clones have been successfully developed for a number of enteroviruses, including poliovirus [21], coxsackievirus B6 [22], coxsackievirus B2 [23], echovirus 5 [24], and enterovirus 71 [25-27], but not for CVA16. In this paper, we report the first construction of an infectious cDNA clone of CVA16. This infectious clone contains the full-length cDNA of CVA16 flanked by a T7 promoter and a poly(A) tail at the 5' and 3' ends, respectively. Transfection of RD cells with RNA transcribed directly from the cDNA clone resulted in the successful recovery of infectious virus. The recovered CVA16 was found to be functionally and genetically identical to its parent strain, and it could be used to facilitate future virological investigation as well as vaccine development for CVA16.

## Results

### Construction of a full-length infectious clone of CVA16

**Figure 1:**
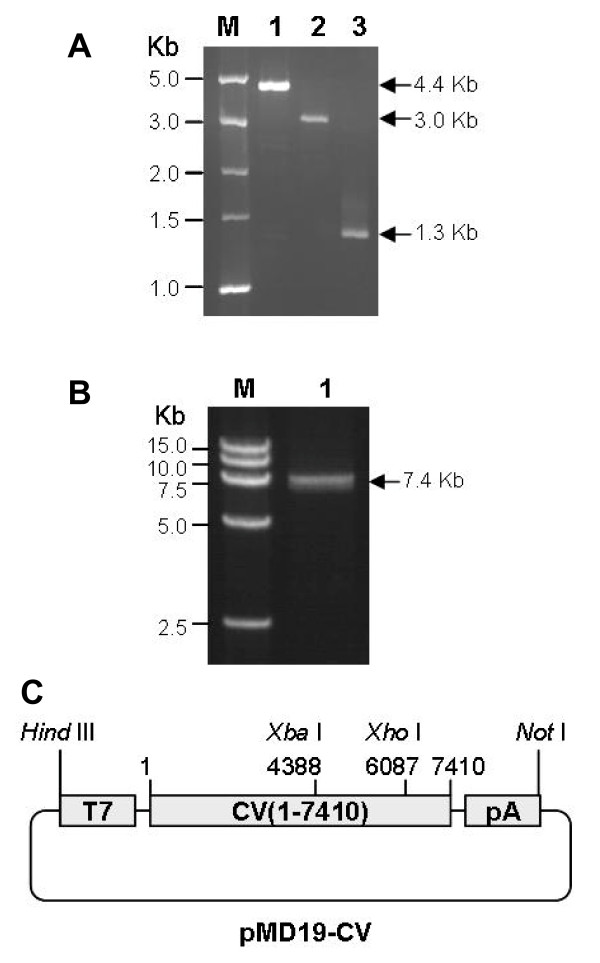
**Construction of a full-length infectious clone of CVA16**. (A) PCR amplification of CVA16 specific fragments. Lane M, DL5000 DNA marker (TaKaRa Biotechnology, Dalian, China); lane 1, CV(1-4392); lane 2, CV(4381-7410); and lane 3, CV(6087-7410-pA). (B) PCR amplification of the CVA16 full-length cDNA plus T7 promoter and 3' poly(A) sequence. Lane M, DL15000 DNA marker (TaKaRa Biotechnology, Dalian, China); lane 1, T7-CV(1-7410-pA) amplicon. (C) Schematic representation of the plasmid pMD19-CV. T7, T7 promoter; CV(1-7410), nucleotides 1-7410 of the CVA16 genome; pA, poly(A) sequence.

The genome of the CVA16 strain shzh05-1 (GenBank: EU262658) is an RNA molecule containing 7410 nucleotides. Viral RNA was extracted and subjected to reverse transcription using oligo(dT) primers. Two overlapping cDNA fragments were amplified from the first strand cDNA, encompassing nucleotides 1-4392 and 4381-7410 of the CVA16 genome, designated as CV(1-4392) and CV(4381-7410), respectively (Figure 1). These two overlapping fragments were then joined via an *Xba*I site at position 4387-4392, and ligated into pcDNA3.1, resulting in the production of pcDNA3.1-CV(1-7410). CV(6087-7410-pA), which contains nucleotides 6087-7410 and a poly(A) tail, was also amplified (Figure 1) and used to replace the corresponding segment within pcDNA3.1-CV(1-7410), thereby yielding pcDNA3.1-CV(1-7410-pA). Sequencing analysis of the pcDNA3.1-CV(1-7410-pA) revealed three nucleotide mutations at positions 2733 (C to T), 2760 (T to C), and 3161 (G to A) within the cDNA when compared with the previously reported sequence (GenBank #EU262658). All three mutations resulted in amino acid changes. The entire cDNA cloning process was repeated, starting from RNA isolation from the same batch of virus. Three clones from two independent cloning events were fully sequenced and the identical mutations were found in all three clones. Thus, these three mutations were not introduced during the cloning process. Instead, they were likely to have been acquired during multiple passage of the virus in cell culture since the original report [28].

To facilitate *in vitro *transcription, a T7 promoter was added upstream of CV(1-7410-pA) by PCR amplification with primers P6 and P7 (Table 1). The resultant PCR product with an expected size of ~7.5 Kb (Figure 1) was cloned into the pMD19-T Simple Vector yielding pMD19-CV, a full-length cDNA clone of CVA16. A schematic representation of pMD19-CV is shown in Figure 1.

**Table 1:** Primers used in this study

Primer	Sequence (5' - 3')	Enzyme site	Purpose
P1	GCCAAGCTTAAAACAGCCTGTGGGTTGTTCCCACCC	*Hind *III	CV(1-4392) amplification

P2	CGGGTCTAGAGCGTAGACTCTTTTGGCTTCAGTC	*Xba *I	CV(1-4392) amplification

P3	CTACGCTCTAGAAAGAAGGA	*Xba *I	CV(4381-7410) amplification

P4	ACAAGCGGCCGCTGCTATTCTGGTTATAAC	*Not *I	CV(4381-7410) amplification

P5	CTTCTCGAGGTTGATTTTGAGCAAGCATTG	*Xho *I	CV(6087-7410-pA) amplification

P6	TATGCGGCCGCTTTTTTTTTTTTTTTTTTTTTTTTT	*Not *I	CV(6087-7410-pA) amplification

P7	CTAAAGCTTAGCTAATACGACTCACTATAGTTAAAACAGCCTGTGGGTTG	*Hind *III	T7 promoter introduction/priming cDNA synthesis from negative-strand RNA

P8	CCTATTGCAGACATGATTGACCAG	none	RT-PCR for negative-strand RNA

P9	TGTTGTTATCTTGTCTCTACTAGTG	none	RT-PCR for negative-strand RNA

Restriction enzyme sites are underlined

### Recovery of infectious CVA16 from the cDNA clone

**Figure 2:**
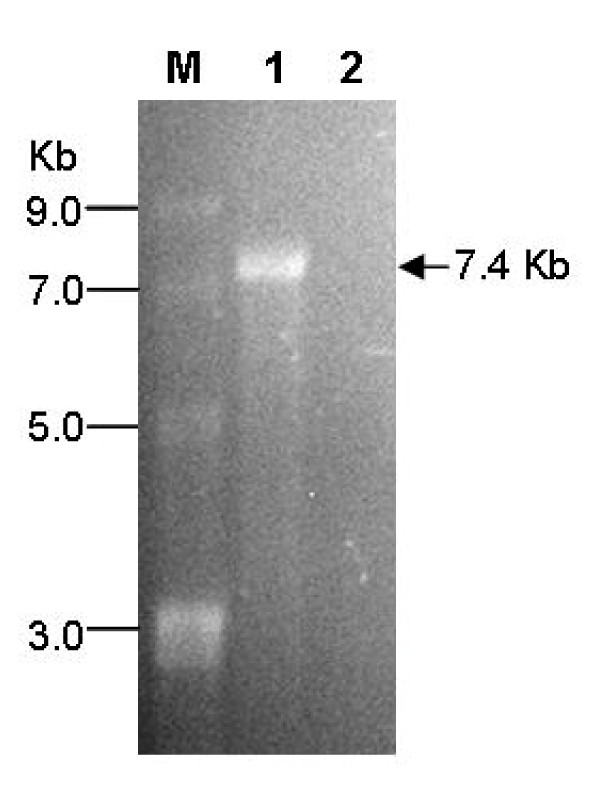
**Analysis of *in vitro *generated RNA transcripts by agarose gel electrophoresis**. *Not*I linearized pMD19-CV was transcribed with or without T7 RNA polymerase. The resultant reaction mixtures were analyzed by electrophoresis on a 1.2% agarose gel. Lane M, ssRNA ladder marker (Cat#N0362S, New England Biolabs); lane 1, reaction mixture with T7 RNA polymerase; lane 2, reaction mixture without T7 RNA polymerase.

PMD19-CV was linearized by *Not*I digestion and used as a template for *in vitro *transcription with T7 RNA polymerase as described in the Materials and Methods. As shown in Figure 2, a ~7.5 Kb band was present in the *in vitro *transcription reaction mixture with T7 RNA polymerase, but not without T7 RNA polymerase, indicating that the band represented RNA transcripts produced from the cDNA clone. The resultant transcripts were used to transfect RD cells. At 72 h post-transfection, cells and supernatants were harvested and analyzed by microscopy and biochemical assays.

**Figure 3:**
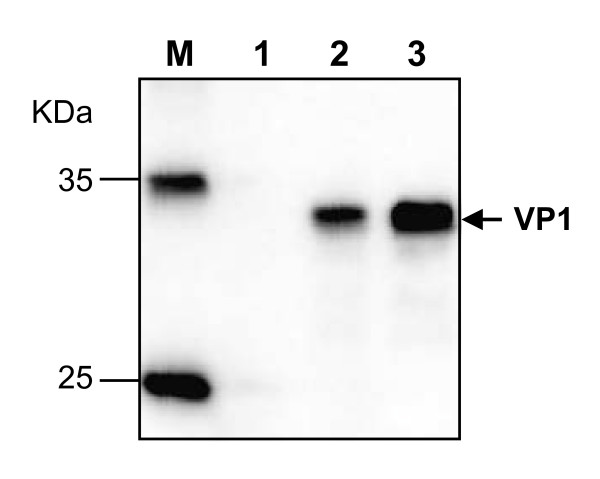
**Detection of VP1 expression in cell lysates by Western blotting**. Protein samples were separated on a 12% polyacrylamide gel and then transferred onto a PVDF membrane. The membrane was probed with a polyclonal antibody against the VP1 protein of CVA16, followed by a corresponding horseradish peroxidase-conjugated secondary antibody. Lane M, protein marker; lane 1, mock-transfected cell lysate; lane 2, RNA-transfected cell lysate; lane 3, wild-type virus infected cell lysate.

Lysates were made from transfected cells and subjected to western blot analysis using a polyclonal antibody against the recombinant VP1 protein of CVA16 to facilitate the detection of viral protein [20]. As shown in Figure 3, a positive signal was not detected in the mock-transfected sample (lane 1), whereas positive bands at ~33KDa were evident in the RNA transfected (lane 2) and the wild-type virus-infected cell lysates (lane 3), indicating the production of correctly processed VP1.

**Figure 4:**
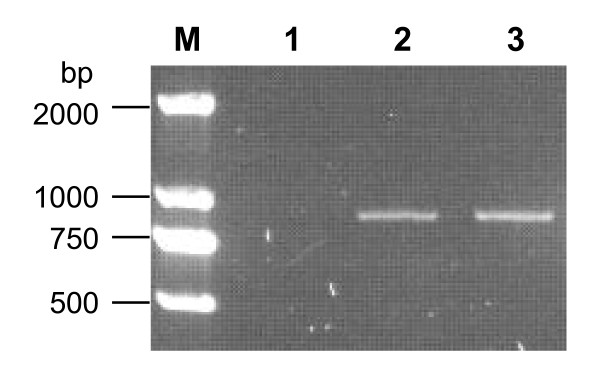
**Detection of negative-sense RNA by RT-PCR**. RNA extracted from transfected or infected cells was subjected to reverse transcription with the primer P7 and subsequent PCR amplification of an 882-bp fragment using primers P8 and P9. Lane M, DNA marker; lane 1, PCR product of mock-transfected cells; lane 2, PCR product of RNA-transfected cells; lane 3, PCR product of wild-type virus infected cells.

The presence of negative-strand viral RNA in the transfected cells was then determined. Primer P7 (Table 1), which is complementary to the negative-strand RNA, was used to prime the synthesis of first strand cDNA, while the primer pair P8/P9 (Table 1) was subsequently used to amplify the nucleotide region 2447-3328. As shown in Figure 4, a PCR product of ~0.9 Kb was observed with both the RNA transfected and wild-type virus-infected samples. In contrast, the negative control (mock transfected sample) did not produce a specific PCR product. This result indicates that the RNA transcript transfected cells synthesized negative-strand viral RNA as did the wild-type virus-infected cells.

**Figure 5:**
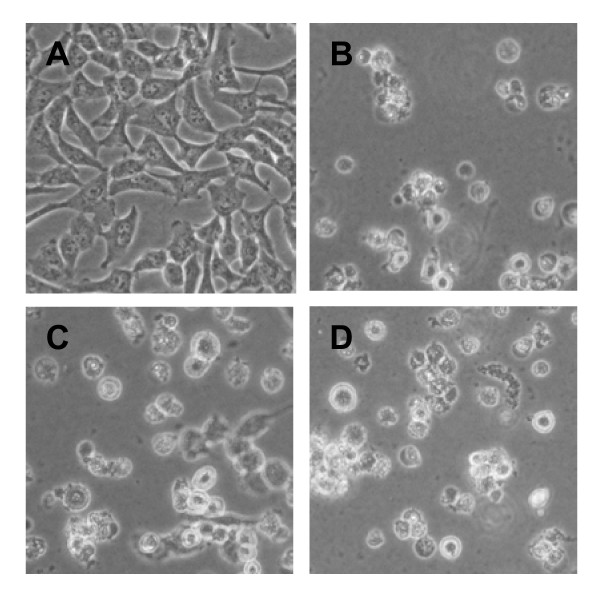
**Phenotypic characteristics of RD cells post-treatment**. (**A**) normal RD cells; (**B**) RD cells infected with wild-type CVA16; (**C**) RD cells transfected with *in vitro-*generated RNA transcripts; (**D**) RD cells infected with recovered CVA16.

The cytopathic effects (CPE) of RNA transfected cells were observed as an indicator of productive virus infection. As shown in Figure 5, the control (mock-transfected) cells appeared to grow normally, whereas the RNA transfected cells displayed typical CPE (including cell rounding, aggregation, and floatation) as did the cells infected by the wild-type virus. Lysates from RNA transfected cells were subsequently used to inoculate RD cells. At 24 ~ 48 h post-inoculation, the lysate-inoculated cells also exhibited severe CPE (Figure 5), indicating that the lysate contained a first generation of recovered virus (designated as R1), which could efficiently infect permissive cells to produce a second generation of recovered virus (designated as R2). The genome of the R1 virus was sequenced and compared to that of the cDNA clone. The sequences were identical (data not shown). Further infection with the R2 virus also caused CPE in RD cells (data not shown). Overall, the above results demonstrate that the RNA transcribed from the CVA16 cDNA clone was capable of generating infectious CVA16.

### Characterization of the recovered CVA16

**Figure 6:**
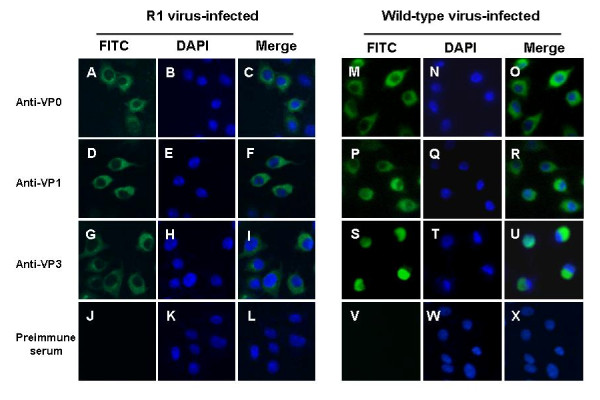
**Immunofluorescence staining of cells infected with the R1 virus or the wild-type virus**. Infected cells were incubated with polyclonal guinea pig anti-CVVP0 (A-C and M-O), anti-CVVP1t (D-F and P-R), anti-CVVP3 (G-I and S-U), or pre-immune serum (J-L and V-X), followed by incubation with a FITC-conjugated goat anti-guinea pig IgG antibody. Cells were also stained with DAPI. (A, D, G, J, M, P, S and V) images captured using a FITC filter; (B, E, H, K, N, Q, T and W) images captured using a DAPI filter; and (C, F, I, L, O, R, U and X) merged images.

Recovered CVA16 was characterized by immunofluorescence. As shown in Figure 6, R1 virus-infected cells were specifically stained using three different anti-CVA16 polyclonal antibodies, but not using preimmune serum. Positive signals appeared to localize in the cytoplasm, which was a similar pattern to that observed for the wild-type CVA16-infected cells (Figure 6). This result indicates that the recovered virus could produce viral proteins specific to CVA16 in a manner indistinguishable from the wild-type virus.

**Figure 7:**
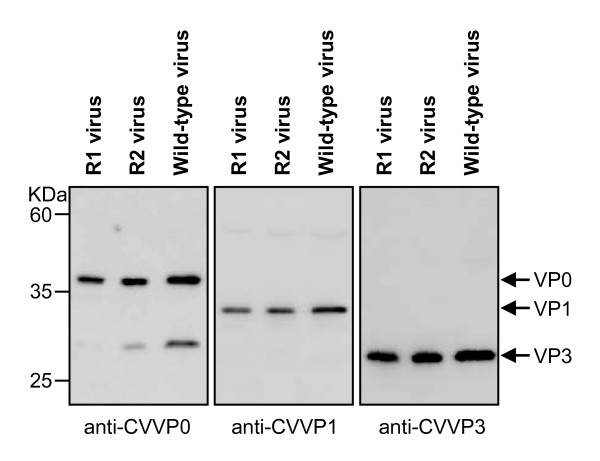
**Western blot analysis of capsid composition of the recovered viruses**. Lysates from cells infected with the R1 or R2 generation of recovered viruses or wild-type virus, were separated by SDS-PAGE, blotted onto PVDF membranes, and probed with polyclonal anti-CVVP0, anti-CVVP1, or anti-CVVP3, followed by incubation with an HRP-conjugated secondary antibody.

The capsid composition of the R1 virus was analyzed by western blotting using the same polyclonal antibodies against VP0, VP1 and VP3 of CVA16. As shown in Figure 7, the R1 virus samples produced positive signals at positions identical to those produced by the parent strain, suggesting no difference in the viral protein expression or processing of both viruses.

**Figure 8:**
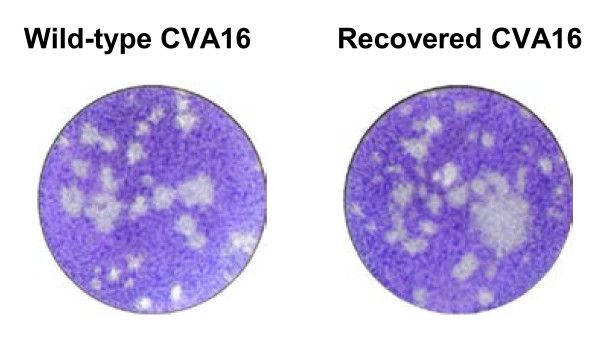
**Plaque phenotype of wild-type and recovered CVA16**. Ten-fold dilutions of virus suspension were inoculated into 24-well plates containing Vero cell monolayers and incubated for 2 h at 37°C. The plaque assay was then performed as described in the Methods section.

The biological characteristics of the wild-type and recovered viruses were also compared. The R1 virus was found to generate the same negative-strand viral RNA as the wild-type virus, as demonstrated by the amplification of a ~0.9 Kb RT-PCR product from the R1 virus (data not shown) and the wild-type virus-infected cells (Figure 4). R1 virus-infected cells were then found to display typical CPE (including cell rounding, aggregation, and floatation) (Figure 5). The R1 virus-induced CPE was indistinguishable from that of the wild-type virus (Figure 5). Moreover, the R1 virus plaque phenotype was similar to that of the wild-type strain (Figure 8).

## Discussion

The aim of this study was to construct an infectious clone of CVA16. The genome of CVA16 is an RNA molecule measuring 7410 bases in length. In our study, viral RNA was reverse transcribed to yield first-strand cDNA, which was then used subsequently as a template for the PCR amplification of CVA16-specific fragments. Two strategies were adopted to obtain a full-length cDNA clone of CVA16. The first was to directly amplify the full-length CV(1-7410) from the reversely transcribed cDNA, while the other was to amplify two fragments, i.e., CV(1-4392) and CV(4381-7410), and subsequently rejoin them via an *Xba*I site, to yield CV(1-7410). The first strategy is successful for the construction of infectious clones of a number of enteroviruses [23,24,29], including the closely related EV71 [27], but it failed for CVA16 in this study (data not shown). However, when we used the latter strategy, we found that CV(1-4392) and CV(4381-7410) could be amplified and subsequently fused to produce CV(1-7410). This suggests that the size of any target fragment is an important factor in the successful amplification of long PCR regions. Interestingly, CV(1-7410) and its slightly longer form, T7-CV(1-7410-pA), were amplified from the cloned plasmid (Figure 1), although it could not be generated from the reverse transcribed first-strand cDNA (data not shown). Given that the reverse transcription reaction mixture was not homogeneous, the purity and/or abundance of the full-length first-strand cDNA could be critical to the successful amplification of full-length double-stranded cDNAs.

*In vitro *generated RNA transcripts were transfected into RD cells via electroporation to regenerate CVA16. The data demonstrates that these RNA transcripts were capable of directing viral protein expression and processing (Figure 3 and 7). It is commonly accepted that negative-strand RNA, together with positive-strand RNA, forms double-stranded replicative intermediates that act as a template for further positive-strand RNA synthesis during RNA genome replication by enteroviruses [30,31]. Thus, the presence of negative-strand RNA was an indicator of efficient viral RNA replication. In this study, negative-strand RNA was detected in RD cells transfected with *in vitro *synthesized positive-strand RNA (Figure 4), indicating that the exogenous RNA transcripts were replication competent. Furthermore, infectious CVA16 virus was recovered from the RNA transcript transfected cells. The resultant recovered virus was detected using CVA16-specific antibodies (Figures 6-7) and it had the same CPE (Figure 5 and 8) as the wild-type virus. Passage of the recovered virus in RD cells consistently led to viral protein expression (Figure 7) and CPE (Figure 5), indicating the infectivity of the recovered virus.

## Conclusions

This study reports the first construction and characterization of a novel infectious cDNA clone of CVA16. This cDNA clone was capable of producing the infectious CVA16 virus, which was genetically and biologically identical to its parent stain. The availability of a CVA16 infectious clone will greatly facilitate the investigation of the genetic determinants of its virulence. This clone will also allow the rapid, rational development and testing of candidate live attenuated vaccines and antiviral therapeutics against CVA16.

## Methods

### Cells and viruses

RD and Vero cells were grown in DMEM (Gibco, Grand Island, NY, USA) supplemented with 10% FBS, 100 U/ml penicillin, and 100 μg/ml streptomycin at 37°C with 5% CO_2_. The CVA16 strain shzh05-1, described in [28], was propagated in RD or Vero cells. Virus titers were determined by microtitration using RD cells and expressed as the 50% tissue culture infectious dose (TCID50), according to the Reed-Muench method [32].

### RNA extraction and reverse transcription

RNA was extracted from CVA16/shzh05-1 infected RD cells using Trizol reagent (Invitrogen, Carlsbad, CA, USA). The extracted RNA was reverse transcribed using oligo(dT) primers and M-MLV reverse transcriptase to produce cDNA (Invitrogen, Carlsbad, CA, USA), according to the manufacturer's instructions. The resultant first strand cDNA was used as a template for subsequent PCR amplification of CVA16 genome fragments.

### Primer design

Primers were designed based on the published sequence of CVA16 strain shzh05-1 (GenBank# EU262658) (Table 1) to amplify specific fragments of the CVA16 genome. Primers P1 and P2 were designed to amplify a cDNA fragment encompassing nucleotides 1-4392, which was designated CV(1-4392), and it also contained engineered *Hind*III and *Xba*I restriction enzyme sites. Primers P3 and P4 were designed to amplify a cDNA fragment encompassing nucleotides 4381-7410, which was designated CV(4381-7410), and it contained engineered *Xba*I and *Not*I restriction enzyme sites. Primers P5 and P6 were designed to amplify a cDNA fragment encompassing nucleotides 6087-7410 with an added poly(A) tail, which was designated CV(6087-7410-pA). Primer P7 contained a *Hind*III site, a T7 promoter sequence, and 20 nucleotides of the 5' UTR of CVA16 cDNA. It was used to introduce the T7 promoter upstream of the full-length cDNA for efficient *in vitro *transcription and to prime the synthesis of first strand cDNA from negative-strand viral RNA. Primer P8 anchored to the nucleotides 2447-2470 of positive-sense CVA16 full-length cDNA while P9 was complementary to the nucleotides 3304-3328 of positive-sense cDNA. Both P8 and P9 were used to detect negative-strand RNA by RT-PCR amplification of a ~0.9 KB fragment (nucleotides 2447-3328).

### Cloning of the full-length cDNA

CV(1-4392) was amplified from the reverse transcribed first strand cDNA using primers P1 and P2 (Table 1). Similarly, CV(4381-7410) and CV(6087-7410-pA) were obtained using the primer pairs P3/P4 and P5/P6 (Table 1), respectively. CV(1-4392) and CV(4381-7410) were digested with *Hind*III/*Xba*I and *Xba*I/*Not*I, respectively, and ligated into *Hind*III/*Not*I digested pcDNA3.1 to produce pcDNA3.1-CV(1-7410). CV(6087-7410-pA) was digested with *Xho*I/*Not*I and then used to replace the corresponding sequence within pcDNA3.1-CV(1-7410), resulting in pcDNA3.1-CV(1-7410-pA). The primer pair P6/P7 (Table 1) was used for PCR amplification with pcDNA3.1-CV(1-7410-pA) as a template to introduce the T7 promoter for *in vitro *transcription. The resultant PCR product containing an engineered T7 promoter sequence upstream of the CV(1-7410-pA) was cloned into the pMD19-T Simple vector (Takara Mirus Bio, Madison, WI, USA), yielding pMD19-CV.

### *In vitro *transcription

PMD19-CV was digested with *Not*I, purified and used as the template for *in vitro *transcription. *In vitro *transcription was performed using the Riboprobe system-T7 *in vitro *transcription kit (Promega, Madison, WI, USA), according to the manufacturer's instructions.

### Transfection

RD cells were grown in T75 flasks to 90% confluency, harvested by centrifugation, then resuspended in OPTI-MEM medium (Cat# 31985, Invitrogen, Carlsbad, CA, USA). Next, 400 μL (4 × 10^6 ^cells) of the cell suspension was mixed with 10 μg of *in vitro *synthesized RNA transcripts. These mixtures were incubated for 3 min at room temperature, transferred into an electroporation cuvette, and then subjected to electroporation at 220 V using the GenePulser Xcell™ electroporation system (Bio-Rad, Hercules, CA, USA). Immediately after electroporation, the mixtures were resuspended in 5 ml of DMEM supplemented with 10% FBS, transferred to a T25 flask, and incubated at 37°C with 5% CO_2 _for 72 h.

### RT-PCR for the detection of negative-strand RNA

Viral RNA was reverse transcribed using primer P7 to detect negative-strand RNA (Table 1). The resultant first strand cDNA was used as a template for PCR amplification of a fragment (nucleotides 2447-3328) with primers P8 and P9 (Table 1). PCR was performed using PrimeSTAR™ HS DNA polymerase (Takara Mirus Bio, Madison, WI, USA) with the following cycle: 94°C for 5 min, followed by 30 cycles at 94°C for 30 s, 55°C for 30 s, 72°C for 60 s, with a final extension of 72°C for 10 min in an MJ Mini™ thermal cycler (Bio-Rad, Hercules, CA, USA).

### SDS-PAGE and western blot analyses

SDS-PAGE and western blotting were performed as previously described [20]. Briefly, proteins were separated on 12% polyacrylamide gels and transferred onto PVDF membranes. Membranes were then probed using one of three home-made CVA16 capsid protein-specific antisera [20], followed by a corresponding horseradish peroxidase (HRP)-conjugated secondary antibody (Sigma, St. Louis, MO, USA). Membranes were developed by chemiluminescence using a BeyoECL Plus kit (Cat# P0018; Beyotime, Shanghai, China) and signals were recorded with a LAS-4000 Luminescent Image Analyzer (Fujifilm Life Science USA, Stamford, CT, USA).

### Immunofluorescence assay

Immunofluorescent staining was performed as previously described [20], using three polyclonal antibodies against the recombinant CVA16 capsid subunit proteins, VP0, VP1, and VP3. Stained samples were examined on an upright fluorescence microscope (Leica, Wetzlar, Germany).

### Plaque assay

The plaque assay was performed using 24-well plates containing Vero cell monolayers. Ten-fold dilutions of virus suspension were inoculated at 400 μl/well and incubated for 2 h at 37°C. The virus suspension was then removed and 1 ml of DMEM containing 2% FBS and 1% low melting point (LMP) agarose (Promega, Madison, WI, USA) was added to each well, before incubating at 37°C. The medium was discarded after several days and cells were fixed in 10% formaldehyde solution then stained with 0.1% crystal violet (Sigma, St. Louis, MO, USA).

## Abbreviations

CVA16: Coxsackievirus A16; HFMD: Hand, Foot, and Mouth Disease; EV71: Enterovirus 71; CPE: CytoPathic Effect; DMEM: Dulbecco's Modified Eagle's medium

## Competing interests

The authors declare that they have no competing interests.

## Authors' contributions

FL, QL and YC performed the experiments. ZH conceived the study and wrote the manuscript. QL participated in the study design and data analyses. All authors read and approved the final manuscript.

## Acknowledgements

We thank Drs Bing Sun and Qi Jin for providing the CVA16 virus. We also thank Dr. Andy Tsun and the International Science Editing for their excellent editorial contribution. This work was supported by a grant (#KSCX2-YW-BR-2) from the Chinese Academy of Sciences "100 Talents" program and a grant (#2010KF-07) from the Biochemical Engineering National Key Laboratory of China. Z.H. gratefully acknowledges the support of SA-SIBS scholarship program.
